# Dielectric Response of Hydrogenated Borophene Monolayers from First-Principles Density Functional Theory Calculations

**DOI:** 10.1021/acsomega.5c12691

**Published:** 2026-04-06

**Authors:** Arpita Varadwaj, Yasunobu Ando, Masahito Niibe, Takahiro Kondo, Iwao Matsuda, Masato Kotsugi

**Affiliations:** † Faculty of Advanced Engineering, 26413 Tokyo University of Science, Tokyo 125-8585, Japan; ‡ Institute of Integrated Research, Institute of Science Tokyo, Yokohama, Kanagawa 226-8501, Japan; § The Institute for Solid State Physics, 13143 The University of Tokyo, Kashiwa, Chiba 277-8581, Japan; ∥ Institute of Pure and Applied Sciences, 13121 University of Tsukuba, Tsukuba, Ibaraki 305-8573, Japan

## Abstract

Considering the incredible impact of 2D materials on emerging technologies, hydrogenated borophene (borophane) has attracted significant interest not only as a tunable platform for electronic and optoelectronic applications but also for its promising catalytic behavior. Here, we present a systematic density functional theory investigation of the structural, electronic, and optical properties of the 6,6 and 5,7 borophane polymorphs with distinct point-group symmetries. The calculated electronic band structures and density of states confirm the semimetallic characteristics of all structures, consistent with previous reports. The complex dielectric function spectra further reveal how structural motifs and hydrogenation patterns influence the onset of optical absorption. For the 5,7 HB monolayers, the first major absorption feature shifts markedly toward the far-infrared region compared with the 6,6 HB, indicating a stronger low-energy electronic response and a more pronounced metallic character. The optical spectra of 6,6 HB also show qualitative agreement with the available experimental UV–vis data. These results provide a comprehensive understanding of how symmetry and bonding topologies govern the electronic and optical behavior of borophane monolayers, offering useful guidance for the design of boron-based 2D optoelectronic materials.

## Graphical abstract

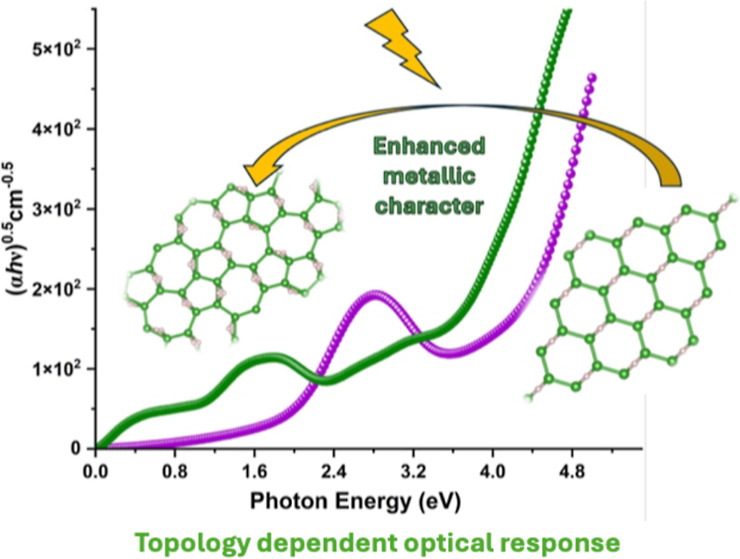

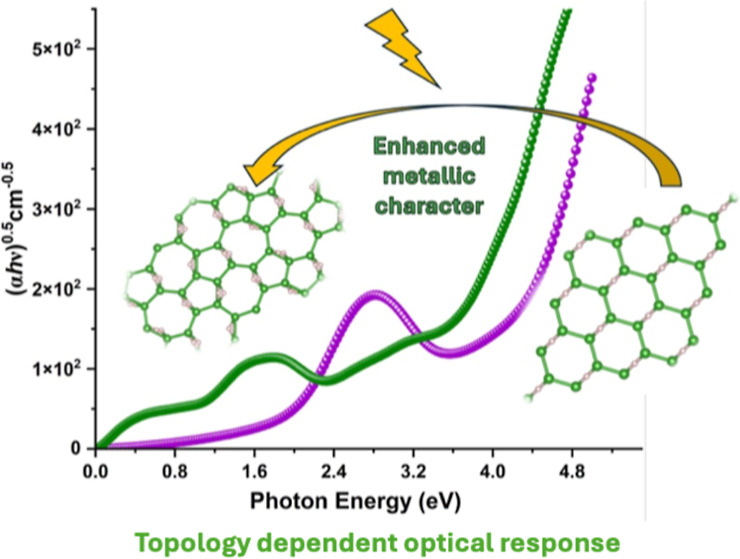

## 1 Introduction

Two-dimensional (2D) materials have gained significant attention for their chemical stability, structural flexibility, and unique optoelectronic properties, which are crucial for advancing modern technologies. Among them, graphene,^1–4^ borophene,^5^ hydrogen boride,^6,7^ MXenes,^8^ and hexagonal boron nitride^9,10^ are the largely studied systems. Hydrogenated borophenes, or hydrogen borides (in short, HB), are hydrogen storage (or hydrogen carrier) systems that are hydrogen-assisted analogues of borophene, called borophanes, with distinct electronic structures and optoelectronic properties.^11,12^

Some variants of HB structures^6^ exist in different phases, each with distinct characteristics, featuring a boron-to-hydrogen stoichiometric ratio of one. For example, they include nanosheets such as 5,7-,^11,13^ 6,6-,^7,14–16^ and 5,6,7-HBs.^11,17^ In these representations, the numbers 5, 6, and 7 indicate the number of boron atoms in the local ring structures. Wang et al.^12,18,19^ have reported new structures of fully hydrogenated borophenes in 2D that are not only phonon-stable but also mechanically robust. These past studies^6,13,16,20^b−^22^ indicate that their geometries, density of states, and band structures^17,23^ may display Dirac nodal features^17,24^ and electron and hole-pockets,^23^ with unique X-ray diffraction patterns,^7^ absorption spectra of B’s K-edge,^13,20,25,26^ high thermal conductivity,^27^ among others.

The 6,6 HB system was synthesized from magnesium diboride (MgB_2_) using liquid exfoliation at room temperature (∼298 K) in a nitrogen environment, employing cation-exchange methods for precise control over their properties.^20,23^ Their geometrical versatility makes them relevant for applications in catalysis,^21^ sensing, and energy storage.^16^ (5–7)-α-Borophene, a symmetric structure derived from TmAlB_4_,^11^ consists of pentagons and heptagons in its α lattice and is classified as a 2D Dirac nodal semimetal, and the two hydrogenated phases of the same system comprise different configurations of the adsorbed H atoms.^13^ The 6,6 HB structure features rings of Dirac nodes that possess a stronger nonlinear electromagnetic response.^24,27^ In contrast, (5,6,7)-γ-HB features a Dirac nodal line; its hydrogenation pattern disrupts the symmetry of the boron framework, leading to a nonsymmorphic HB sheet.^17^ Reports also include armchair-like (5,5) and zigzag-like (10,0) boride hydride nanotubes (BNTs), designated as (5,5) H-BNTs and (10,0) H-BNTs, respectively.^28^ Thus, designing new graphene-like hydrogenated boron structures in 2D that display Dirac cones or rings^24^ may involve arranging B_2_H_6_ molecules into a periodic array in an infinite crystal.

Tateishi et al.^11^ reviewed the work of Niibe et al.,^13^ who have reported the density functional theory calculated energies of σ/σ* and π/π* bands for the 5,7-α_1_-borophene (5,7-α_1_-B_16_H_16_) and 5,7-α_2_-borophene (5,7-α_2_-B_16_H_16_) HB sheets, comparing these findings with spectral features measured using soft X-ray emission and absorption (SXE and SXA) spectroscopy.^11,13^ They have linked the spectral peaks and the partial density of states (PDOS)-based orbital-energy curves coupled with the valence and conduction bands and concluded that their spectroscopic results align very well with the calculated PDOS orbital-energy features of σ/σ* and π/π* bands for 5,7-α_1_ HB, providing evidence of semimetallicity linked to a Dirac nodal loop near the Fermi level.

Recent experimental and theoretical studies^12,16,29^ have demonstrated the tunability and functional potential of hydrogenated borophene (HB) sheets. For 5,7-HB, chemical exfoliation of YCrB4 crystals has yielded multiple HB sheet types with distinct hydrogenation patterns, exhibiting semimetallic, partially suppressed, or insulating electronic states.^30^ Hydrogenation stabilizes the 5,7-boron network and strongly influences the electronic structure, highlighting the role of precise hydrogen placement in controlling material properties. In particular, three types of 5,7-HB sheets were obtained from YCrB4 via liquid exfoliation and ion-exchange reactions; however, the exact hydrogenation patterns (α_1_ and α_2_ configurations) remain unresolved, and the insulating behavior is observed only in sheets that undergo partial oxidation. On the other hand, 6,6-HB sheets have been explored on nickel substrates, where thermal treatment up to 300 °C promoted hydrogen desorption, enhanced structural stability, and facilitated the formation of nickel boride layers at the borophane–substrate interface.^31^ These findings demonstrate how hydrogenation patterns and substrate interactions can modulate both stability and the optoelectronic response, providing valuable guidance for designing functional HB-based materials.

In this study, we expand on the investigation of 5,7 HB monolayer structures, focusing on the geometry, electronic, and optical properties of two specific hydrogenation patterns: 5,7-α_1_-B_16_H_16_ and 5,7-α_2_-B_16_H_16_ with orthorhombic *Pbam* geometry. We employ density functional theory (DFT) for our analysis, utilizing periodic boundary condition calculations primarily at the Perdew–Burke–Ernzerhof (PBE) level.^32,33^ While earlier studies established the semimetallic band topology and structural stability of these HB sheets, a comparative understanding of how different hydrogenation patterns influence their anisotropic optical and dielectric behavior remains limited. To enhance our understanding of the optical absorption (dielectric and Tauc) characteristics,^6,13,15^ we examine the projected density of states (PDOS), band structures, and optical dielectric spectral features for systematic comparison, the latter evaluated using the Kubo-Greenwood formalism.^34,35^ We utilize the PBE and PBEsol functionals to demonstrate consistency in results and to reveal variations in the onset of optical absorption relative to the theoretical level applied and available experimentally.^20^ The present work therefore aims to clarify the relationship between bonding topology, semimetallic electronic structure, and polarization-dependent optical response in these polymorphs and to assess the robustness of the predicted properties with respect to functional choice and structural configuration.

## 2 Computational Methods

Monolayers of 6,6- and 5,7-hydrogen borides (HBs) in their respective point group symmetries (vide infra), respectively, were constructed and fully relaxed using the nonrelativistic electron exchange and correlation functional called Perdew–Burke–Ernzerhof (PBE).^32,33^ For the 6,6 HB lattice, which was taken from ref 24 and from the crystal of MgB_2_,^36^ the Brillouin zone integration was performed with a Γ-centered *k*-point mesh of 17 × 9 × 1 and 21 × 21 × 1, respectively. Methfessel–Paxton smearing^37^ was employed, using a width σ of 0.1, suitable for semimetallic systems. The 5,7 borophene structures were derived from the crystal structure of YCrB_4_ ^36^ to create the hydrogenated analogues 5,7-α_1_ and 5,7-α_2_. This was achieved by strategically introducing hydrogen atoms at specific positions within the B–B bonding regions of the 5- and 7-membered boron rings. The *k*-point meshes were adjusted for the 5,7-α_1_, and 5,7-α_2_ structures to 4 × 9 × 1. A plane wave energy cutoff of 520 eV was set, with convergence criteria for total energy and force per ion at approximately 10^–8^ eV and 0.005 eV/Å, respectively. Spin polarization was included in all calculations, along with the conjugate gradient algorithm^38^ to verify the magnetic ground state. All investigated hydrogenated borophene configurations converged to nonmagnetic solutions, with no residual magnetic moments and identical total energies between spin-polarized and nonspin-polarized calculations within numerical accuracy. Therefore, the results discussed in the manuscript correspond to the nonmagnetic ground state. Additional test calculations were performed using the SCAN *meta*-GGA functional as well as PBE-level calculations, including vdW dispersion corrections. These tests produced only minor quantitative differences in structural parameters and spectral intensities and did not alter the semimetallic character or the relative optical trends.

Dynamical stability of the optimized structures was examined using phonon dispersion calculations within the density functional perturbation theory (DFPT)^39^ framework as implemented in VASP and interfaced with the Phonopy package.^40^ In addition, finite-temperature stability was assessed by performing ab initio molecular dynamics (AIMD) simulations^41,42^ in the NVT ensemble at selected temperatures for 10 ps with a time step of 1 fs. These calculations were used to verify the structural robustness of the hydrogenated borophene sheets under thermal fluctuations.

The density of states (DOS) was computed using a high-density *k*-point mesh of 31 × 31 × 1 that invoked the Tetrahedron method with Blöchl corrections,^43^ whereas the band structures were calculated at the high-symmetry *k*-points as conventionally employed for 2D materials, but Gaussian smearing was chosen. To avoid intermolecular interactions between adjacent layers, a vacuum of 15 Å was applied to the monolayer.

The optical dielectric spectra were evaluated computing the imaginary part of the dynamic dielectric function ε^*i*^_αβ_(ω), which reflects the absorption behavior (an inelastic process)^44,45^ and is the integral part of the complex dielectric function ε­(ω), where ε (ω) second rank tensor given by ε­(ω) = ε_1_ + *i*ε_2_ *=* ε^*r*^_αβ_(ω) + *i*ε^i^_αβ_(ω), and ε_1_ = ε^*r*^_αβ_(ω) represents the real part and is related to electronic polarizability of the material^27,44^ (an elastic process) via the Clausius–Mossotti relation^46–48^ and explains light polarization.^44^ We have done so because the dielectric function in the long-wavelength limit (*q* = 0) is directly proportional to the absorption spectrum.^49,50^ The popular Kubo-Greenwood formula^34,35^ was utilized to evaluate ε^i^(ω), whereas Kramers–Kronig transformation was used to extract the real part of the dielectric function ε^*r*^(ω) from the ε_2_(ω). A relatively dense *k*-point mesh of 10 × 10 × 1 was employed for these calculations for 5,7-borophane and 21 × 21 × 1 for 6,6 HB lattice for the evaluation of the frequency-dependent electronic (optical) contributions to the relative dielectric permittivity ε^*r*^ by the relationship given by ε^*r*^ = ε­(ionic) + ε­(optic), in which the latter involved the inclusion of a large number of unoccupied bands for convergence. Since these calculations are computationally expensive, and the cell of 5,7-borophane contains 32 atoms in the cell, computationally affordable 10 × 10 × 1 *k*-point density was used. The density functional perturbation (DFPT)^39^ was invoked. The effect of spin–orbit coupling is not considered, since the chemical systems investigated do not comprise heavy atoms.

The electron localization function (ELF)^51–53^ was calculated from the converged charge density to analyze bonding characteristics and charge localization in the hydrogenated borophene sheets. The resulting ELF isosurfaces and planar maps were visualized to identify the nature of B–H–B bonding and electron distribution. All calculations were performed using the Vienna Ab initio Simulation Package (VASP).^42,53^

## 3 Results and Discussion

### 3.1 Lattice and Geometry of Borophanes

The four hydrogen boride (HB) structures reported previously using theoretical methods (combined particle-swarm optimization and DFT-PBE methods^22^) include the following: (i) The ladder-like cluster–extended HB layers (space group *C2/m*), (ii) the buckled B_7_-cluster-based extended HB sheets (*Pbcm*), (iii) the hydrogenated graphene-like boron monolayers (*Cmmm*), and (iv) the two-dimensional hydrogenated borophene (*Pmmn*). In the present study, we focus on the *Cmmm* structure of 6,6 HB and the *Pbam* structures of 5,7 HB. The 5,7-HB configurations are represented by two distinct structural variants, denoted as α_1_ and α_2_, both possessing *Pbam* symmetry. These variants differ primarily in the arrangement of hydrogen atoms around the 5- and 7-membered boron rings, which leads to subtle differences in their bonding characteristics and electronic behavior. Our optimized structures are in excellent agreement with previously published results,^17,23,24^ confirming the reliability of our computational approach. Table 1 compares the lattice properties of the unit cells for the 5,7- and 6,6-HB monolayers, and Figure 1a–c illustrates their optimized geometries. In all cases, the two-dimensional boron framework is sandwiched between two layers of hydrogen atoms, which decorate both sides of the B–B bonding regions while preserving the overall 2D architecture. The hydrogen atoms are predominantly concentrated near the B–B bonds of the 5- and 7-membered boron rings in the 5,7-HB monolayers and near the 6-membered rings in the 6,6-HB monolayer.

**Table 1:** PBE-Optimized Lattice Constants (a, b, α, β, and γ), and Energy/Atom for Different Polymorphs of 5,7- and 6,6 HB Monolayers

crystal system	formula unit	*a* (Å)	*b* (Å)	α (deg)	β (deg)	γ (deg)	energy/atom (eV)
5,7-α_1_ HB	B_16_H_16_	11.295 (11.292)^a^	5.820 (5.875)^a^	90	90	90.0	–4.81149
5,7-α_2_ HB	B_16_H_16_	11.300 (11.301)^a^	5.810 (5.829)^a^	90	90	90.0	–4.83287
6,6 HB	B_4_H_4_	2.988 (3.018)^a^ ^,^ ^b^	5.299 (5.287)^a^ ^,^ ^b^	90	90	90.0	–4.86968
6,6 HB	B_2_H_2_	3.046	3.046	90	90	120.6	–4.96628

a Values of *a* and *b* in brakets taken from ref 17 that utilized the PBE functional implemented in Quantum Espresso code.^54^

b Values of *a* and *b*, 2.988 (3.018) vs 5.299 (5.290) Å, in refs 24 and 27.

**Figure 1:**
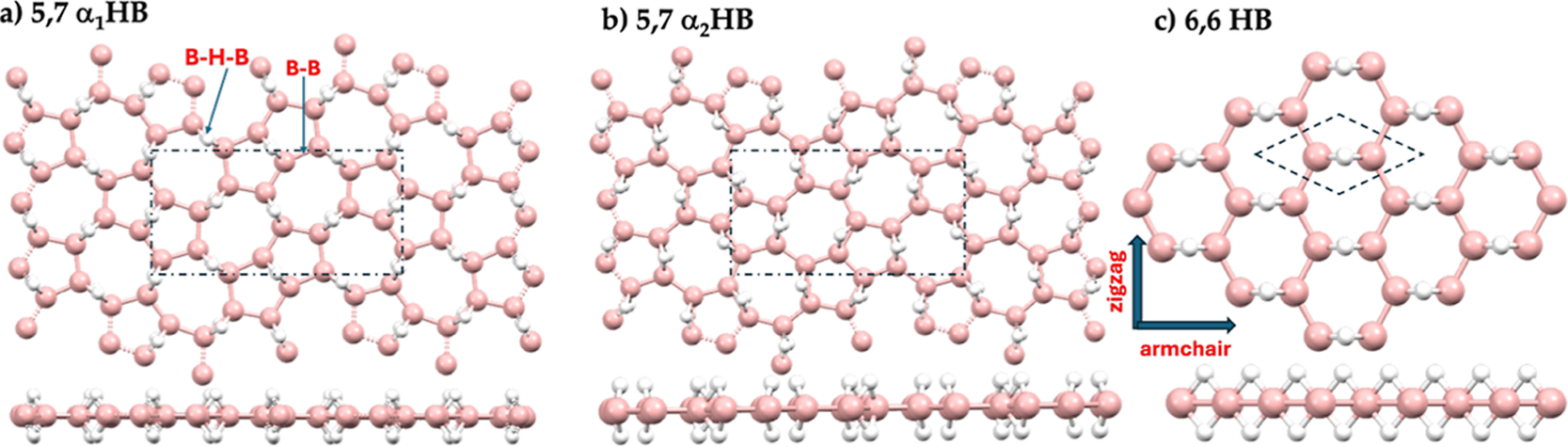
Comparison of PBE-optimized polymorphs of (a,b) 5,7- and (c) 6,6-HB lattice structures. See Tables 1 and 2 for geometric details of these lattices. Figures in the upper and lower panel in each entry refer to the top and side views, respectively. Zig-zag and arm-chair directions are marked only in (c) for 6,6-HB. B and H atoms as large and tiny balls are colored peach and white, respectively.

Table 1 lists the details of the lattice properties (viz. the lattice constants *a*, *b*, α, β, and γ), which vary depending on the lattice type and the nature of the atomic compositions involved. The corresponding properties computed using PBEsol are provided in Table S1 of the Electronic Supporting Information (ESI). For 6,6 HB, both the conventional cell (B_4_H_4_) and the primitive unit cell (B_2_H_2_) were optimized, and the obtained lattice constants are in good agreement with previous reports. Specifically, for the conventional 6,6 HB structure, the lattice constants are *a* = 2.99 Å and *b* = 5.30 Å, which compare reasonably well with earlier studies.^20,23,24^ In the case of the primitive B_2_H_2_ unit cell, the optimized lattice constants are *a* = *b* = 3.046 Å with γ = 120.6°. Some earlier studies reported γ ≈ 60°,^18,55^ which corresponds to an equivalent rotated primitive cell representation of the same hexagonal lattice. Notably, the crystallographic axes *a* and *b* in ref 27 for 6,6 HB were interchanged relative to those reported by Jiao et al.;^24^ however, our choice of crystallographic axes where the zigzag and armchair directions correspond to *a* and *b*, respectively, is consistent with those used by both Tateishi et al.^23^ and Jiao et al.^24^ The smaller B_2_H_2_ unit cell was subsequently adopted for further electronic and optical property calculations.

For the 5,7 HB, the α_1_ polymorph is approximately 0.0214 eV less stable than the α_2_ polymorph. The energetic stability of α_2_ over α_1_ agrees well with the relative energy stability of 0.021 eV reported by Cuong et al.,^17^ despite very marginal discrepancies in the lattice constants a and b between our findings and those in ref 17. The aforesaid discrepancy is not very unreasonable since calculations performed using VASP are likely to differ from those of Quantum Espresso,^54^ in that, we used the PAW potential rather than the ultrasoft pseudopotential, and different cutoff criteria.

In each lattice, two distinct types of B–B bonds are present: *r*(B–B) and *r*(B–B)_b_ (see Table 2, Figures 1 and S1 (ESI)). The B–B bonds without hydrogen bridging are shorter and exhibit electron localization, which plays a crucial role in determining the geometry, akin to covalent bonding. In contrast, the B–B bonds that include bridging hydrogen atoms are influenced by electron localization around the hydrogen atoms in the bonding region. The same feature was previously observed for 6,6 HB, in which B–B bond distances without and with hydrogen bridging were ca. 1.721 Å and 1.812 Å, respectively, and the B–H bond distance was ca. 1.326 Å.^27^ The B–B–B and B–H–B angles vary as one move from one ring to another in each of the two 5–7 HB rings and differ among the three lattices of 6,6 HB, thereby confirming geometrically distinct phases.

**Table 2:** Selected PBE-Level Average Intranuclear Atom–Atom Bond Distances (Å) and Bond Angles (degrees) of the Studied Polymorphs of 5,7 and 6,6 HB Monolayers.^a^

system	boron ring type	*r*(B–B)	*r*(B–B)_b_	*r*(B–H)	∠B–B–B	∠B–H–B
5–7 HB-α_1_	B_5_-ring	1.731 ± 0.029	1.832 ± 0.0	1.321 ± 0.0	108.0 ± 2.1	87.8 ± 0.0
	B_7_-ring	1.731 ± 0.029	1.790 ± 0.029	1.322 ± 0.009	128.6 ± 8.9	85.4 ± 0.8
5–7 HB-α_2_	B_5_-ring	1.716 ± 0.028	1.814 ± 0.069	1.333 ± 0.014	108.0 ± 5.2	85.5 ± 3.1
	B_7_-ring	1.726 ± 0.015	1.812 ± 0.072	1.333 ± 0.014	128.6 ± 5.6	85.5 ± 3.1
6,6 HB (*Cmmm*)	B_6_-ring	1.712 ± 0.0	1.812 ± 0.0	1.326 ± 0.0	120.0 ± 1.1	86.2 ± 0.0

a The subscript “b” in (B–B)_b_ refers to the B–B bond that contains bridging H atoms.

To further confirm the stability of the optimized HB sheets, we examined their dynamical and thermal stability. The calculated phonon dispersions exhibit no imaginary frequencies throughout the Brillouin zone, indicating that the structures correspond to dynamically stable minima.

In addition, AIMD simulations were performed in the NVT ensemble at finite temperatures of 300–700 K for 10 ps with a time step of 1 fs. During the simulations, the frameworks remain structurally intact and exhibit only small thermal fluctuations at equilibrium positions. These results collectively confirm the structural robustness of the investigated HB configurations. The phonon dispersions and AIMD results are provided in the Supporting Information (see Figure S1–S4).

### 3.2 Density of States (DOS) and Electronic Band Structures of Borophanes

The PBE-level PDOS of 5,7-α_1_ and 5,7-α_2_ HB (Figure 2a,b, left) shows that the highest occupied and lowest unoccupied states overlap at the Fermi level (*E*_F_ = 0 eV), providing evidence of a finite density of states at *E*_F_ and confirming the gapless, semimetallic nature of these systems, as observed previously for borophene monolayer^56^ and borophene oxide B_2_O in 2D.^57^ In 5,7-α_1_ HB, the B 2s states dominate the energy range from −8 to −2 eV, with a pronounced peak near −6 eV, whereas the in-plane B 2p_*x*_ and 2p_*y*_ orbitals govern the states near *E*_F_. Similar trends are observed for 5,7-α_2_ HB. The σ (σ*) and π (π*) states arise from the hybridization of in-plane (p_*x*_, p_*y*_) and out-of-plane (p_*z*_) orbitals, respectively, with 2p_*x*_/2p_*y*_ states playing a key role in shaping the local electronic structure at the Fermi level, as indicated by Jiao et al.^24^ for the *C2*_/_m lattice of 6,6-HB. While previous studies^58^ reported only total PDOS for these phases, our analysis provides detailed orbital-specific contributions for both occupied and unoccupied states, in agreement with Niibe et al.^13^

**Figure 2:**
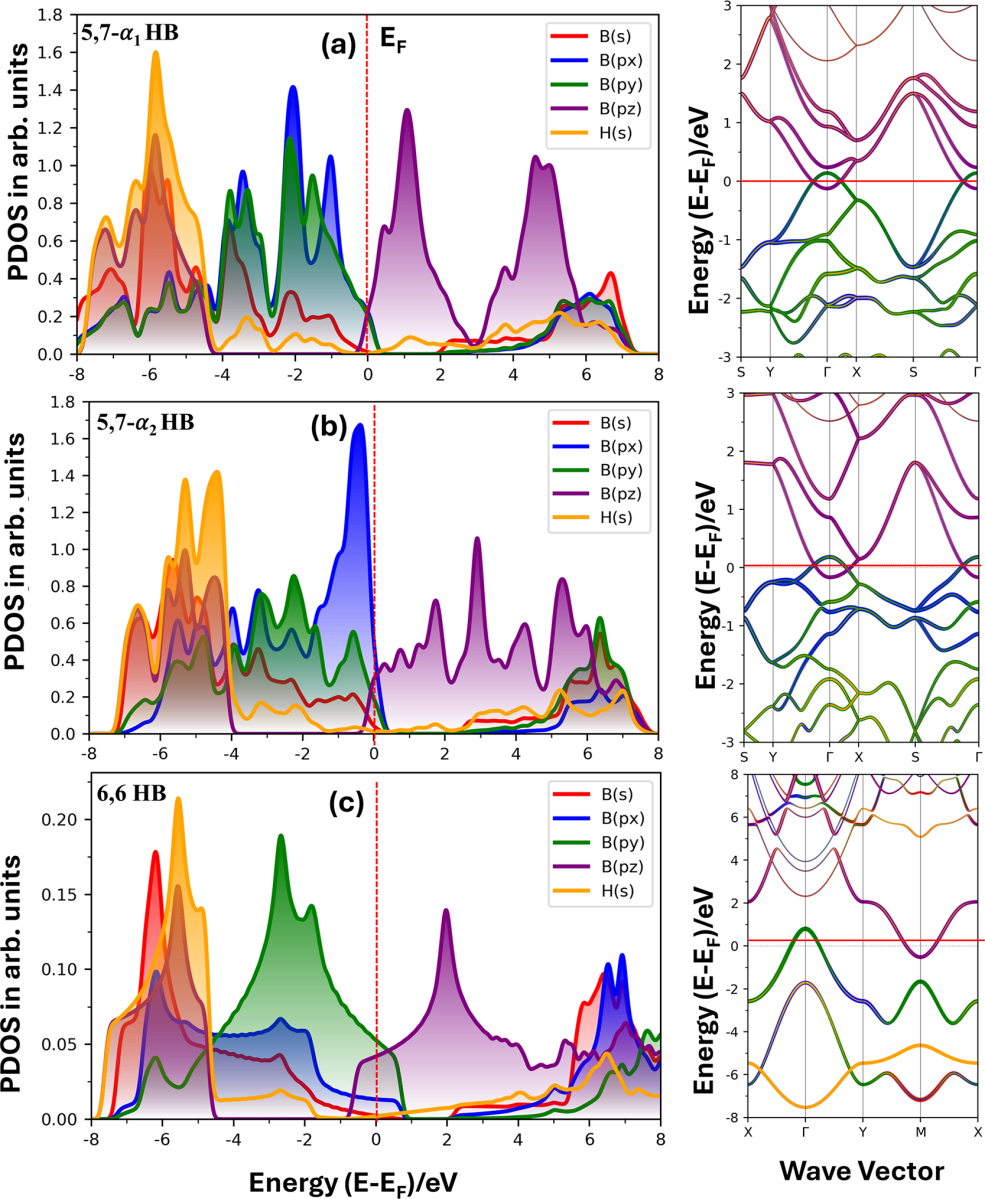
Comparison of PBE-level (left) partial density of states (PDOS) and (right) electronic band structure of (a) 5,7-α_1_, (b) 5,7-α_2_, and (c) 6,6-HB (lattices). The Fermi level, *E*_F_, marked at 0.0 eV in (a) is also applicable to (b,c). The color code used for PDOS for each atom (and orbital) type is the same as that of the band structure.

The electronic band structures of the HB systems indicate that both 5,7-α_1_ with 5,7- α_2_ HBs exhibit Dirac nodal loops (Figure 2a,b right), primarily arising from p’s orbital states. These nodes are located at and near the center of the Brillouin zone, Γ­(*k* = (0, 0, 0)), for 5,7-α_1_ and 5,7- α_2_,^58^ monolayers (see Figure 2a,b), aligning with previous findings.^17,24^ Indeed, these characteristics contrast sharply with those reported for 6,6 and 5,7-borophene, where the Fermi level exhibited significant occupation by conduction and valence band states emerging from different energy level of orbital states, and no discernible Dirac features were observed.^24^ For borophene, the energy band crosses the Fermi level along the Γ-X path, in the vicinity of the X-point.^59^ In contrast to pristine borophene polymorphs, where metallicity originates from strongly dispersive in-plane boron networks with multiple band crossings at the Fermi level, hydrogenation reorganizes the electronic structure through the formation of multicenter B–H–B bonds. This modification reduces the number of band crossings and stabilizes nodal-loop-type semimetallic features near the EF in the present HB lattices, highlighting the role of hydrogenation in tuning both the topology and anisotropy of the electronic structure.

In contrast to the 5,7-α_1_ and 5,7-α_2_ lattices, the electronic structure of 6,6-HB (Figure 2c, right, does not exhibit any Dirac nodal loops. While the conduction band minimum (CBM) crosses the Fermi level and reaches the valence band maximum (VBM) for this system, indicating their semimetallic nature, a direct band gap may be noticeable at the high-symmetry Γ and M points. Previous reports on the 5,6,7-γ_2_ model perhaps exhibits a tiny bandgap,^17^ but in this case, the CBM at the Γ-point touches the Fermi level and the VBM at the same point is below the Fermi level. This attribute may be reminiscent of observations in the 5,6,7-γ_2_, 5,6,7-γ_3_ and 5,6,7-γ_4_ monolayer lattices, where Dirac nodal loops were also absent and the authors of ref 17 assigned 5,6,7-γ_2_ as an insulator and 5,7-α_1_, 5,7-α_2_, and 5,6,7-γ_1_ borophanes as semimetals based on the topological index *Z*_2_ that differentiates semimetals from insulators. In the case of borophene,^59^ a direct bandgap of about 2 eV was suggested along the Γ-Y direction caused by the direct interband transitions involving high-energy hot electrons. This indicates a compensated semimetallic state with a finite density of states at the *E*_F_ rather than a nodal-loop semimetal. Such behavior differs from pristine borophene, where multiple dispersive bands cross *E*_F_ along extended k-paths, reflecting its strongly metallic character. Hydrogenation therefore reorganizes the electronic topology, leading to nodal-loop semimetallicity in 5,7-HB and a compensated semimetallic state in 6,6-HB. Nevertheless, it was suggested using DFT calculations that biaxial or uniaxial loading along the zigzag direction of B_1_H_1_ can lead to bandgap opening, with a bandgap of 1.6 eV.^27^

For 5,7-α_1_, or 5,7- α_2_, on the other hand, we noted the existence of a hole pocket around the Γ-point and an electron pocket around the S-point. This pattern in the difference of the position of the pockets reflects the influence of the high-symmetry *k*-points utilized in this and other study,^23^ but the topology of the band dispersion is virtually similar. An earlier study^60^ suggested the semiconducting nature of both δ_3_ and δ_5_ borophanes with indirect band gaps of 1.51 and 1.99 eV, respectively, which may be ideal for applications in nanoelectronics. Our investigation, however, aligns with both experimental and previously reported theoretical results, confirming that all lattices examined in this study are indeed semimetallic. We also note that calculations using PBEsol yield essentially a similar band structure and DOS as PBE, confirming the robustness of the electronic features. These semimetallic and nodal-loop features near *E*_F_ are expected to influence the low-energy dielectric response through both interband transitions and possible intraband contributions, as discussed in the Optical Dielectric Constant and Dielectric Dispersion of Borophanes section.

### 3.3 Electron Localization Function (ELF)

To elucidate the real-space bonding characteristics underlying the electronic and optical anisotropy of the hydrogenated borophene (HB) monolayers, we analyzed the electron localization function (ELF)^61,64^ for the 5,7-α_1_, 5,7-α_2_, and 6,6 HB structures (Figure 3). For the latter, a 2 × 2 × 1 supercell was used. The ELF provides a direct measure of electron pairing and localization, enabling visualization of multicenter bonding interactions that are not readily captured from band structure or projected density of states alone.

**Figure 3:**
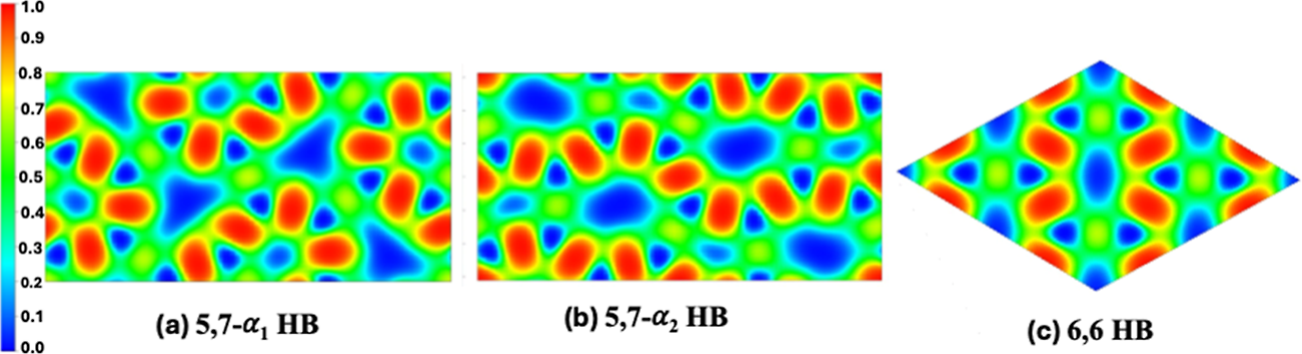
Electron localization function (ELF) distributions for hydrogenated borophene monolayers.

For all HB lattices examined, the ELF maps reveal pronounced electron localization in the regions between boron and hydrogen atoms, forming characteristic 3c–2e B–H–B bonds. These bonds exhibit a banana-like distribution of electron density that is displaced slightly out of the boron plane, reflecting the multicenter σ-type bonding analogous to that observed in diborane-type systems.^18^ Notably, hydrogen atoms are positioned on both sides of the boron framework, producing an out-of-plane distribution of localized charge that breaks strict planar symmetry while preserving the overall lattice periodicity.

In the 5,7-α_1_ and 5,7-α_2_ HB lattices, the ELF distribution along the B–H–B bridges is spatially continuous and forms extended localization channels connecting adjacent boron atoms. This connectivity supports delocalized in-plane electronic pathways while maintaining partial charge localization along the bridging hydrogen direction. The resulting bonding topology is consistent with the nodal-loop semimetallic electronic structure identified in the band analysis, where in-plane B p_*x*_/p_*y*_ orbitals contribute to dispersive states near the Fermi level, while the out-of-plane component associated with the B–H–B bridges modulates orbital hybridization and contributes to the observed anisotropic response.

The 6,6-HB lattice shows a similar multicenter bonding motif; however, the ELF distribution is comparatively more symmetric around each bridge, reflecting the higher structural uniformity of the six-membered ring network. In addition to the B–H–B bridges, this lattice also contains direct B–B bonds without hydrogen bridging, for which the ELF maxima are located at the bond centers, consistent with conventional covalent B–B σ bonding reported in previous studies.^27^ Although the conduction and valence bands cross the Fermi level at different *k*-points, producing a compensated semimetallic state, the ELF still indicates that the electronic structure is governed by the same out-of-plane three-center bonding framework. This suggests that differences in nodal features between the polymorphs arise primarily from lattice symmetry and orbital overlap rather than changes in the fundamental bonding character.

Overall, the ELF analysis confirms that hydrogenation reorganizes the boron electronic network into a quasi-planar framework interconnected by out-of-plane three-center bonds. This bonding topology stabilizes the semimetallic electronic structure and provides a structural basis for the anisotropic optoelectronic response discussed in the following section.

### 3.4 The Optical Dielectric Constant and Dielectric Dispersion of Borophanes

Static dielectric constants were obtained by using DFPT, while the frequency-dependent optical spectra were computed within the independent-particle approximation by using the Kohn–Sham electronic structure. Both PBEsol and PBE functionals were applied to all the lattices to ensure consistency and reproducibility, and the former method has proven to be more accurate than the latter method in producing experimental dielectric constants and phonon modes for bulk systems.^62^ The electronic (optical) dielectric constants for all three lattices are presented in Table 3, highlighting the dominance of specific polarizations that vary with space group. For each case, the three diagonal components differ from each other either marginally or markedly, meaning that they are inequivalent with ε_*xx*_ ≠ ε_*yy*_ ≠ ε_*zz*_. In particular, the in-plane component (ε_||_, along *xx*- and *yy*-directions) is greater than the out-of-plane component (ε_⊥_, along *zz*-direction) for all monolayers, analogous to what was revealed by Mortazavi et al.^27^ for 6,6 HB. The macroscopic optical response is governed by the high-frequency (ion-clamped) dielectric response (ω → ∞), where only the electronic subsystem responds while the ions remain fixed.^63^

**Table 3:** Comparison of *xx*-, *yy*-, and *zz*-Polarized and Average Relative Dielectric Permittivity of Three Different Monolayers of HB

HB system		PBEsol				PBE		
	ε_*xx*_	ε_*yy*_	ε_*zz*_	ε_mean_	ε_*xx*_	ε_*yy*_	ε_*zz*_	ε_mean_
5,7-α_1_(*Pbam)*	2.19	2.09	1.62	1.97	2.03	1.92	1.62	1.86
5,7-α_2_(*Pbam)*	2.85	2.49	1.62	2.32	2.61	2.32	1.61	2.18
6,6 HB (*Cmmm*)^a^	1.72 (1.72)	1.92 (1.96)	1.64 (1.62)	1.76	1.70	1.91	1.62	1.74
6,6 HB (*Pm*)^b^	1.54	1.69	1.47	1.57	1.53	1.68	1.47	1.56

a Parentheses values are taken from ref 27 that used PBE level of theory in conjunction with DFPT.

b The B_2_H_2_ lattice (formula unit) of Ta et al.^55^ was fully optimized in this work using PBEsol and PBE (*a* = 3.006 Å; *b* = 3.044 Å) and used for the evaluation of the dielectric function.

Ta et al.^55^ have recently reported absorption characteristics using the imaginary part of the polarizability (Imα­(ω) (in Å)) rather than using the complex dielectric function, where *Im* refers to the imaginary part of polarizability tensor α­(ω), and ω represents the frequency. This means that they did not elucidate the nature of the external electric field response that can be revealed through the real part of the dielectric function. We fully relaxed their monoclinic lattice (space group *Pm* (space group no. 6)) with a *k*-point grid of 21 × 21 × 1 (same as that used for the *Cmmm* monolayer), and our calculation yielded PBEsol (PBE) values of 1.54 (1.53), 1.69 (1.68), and 1.47 (1.47) for ε_*xx*_, ε_*yy*_, and ε_*zz*_, respectively. The small difference between these values with respect the corresponding ones (Table 3) is emanated from the underlying difference in the lattice constants, in which, the lattice of Ta et al.^55^ encompasses a γ value of 60° that is markedly different from α and β (α = β = 90°). This demonstrates further that a marginal change in the honeycomb-like hexagonal 6,6 HB lattice and its symmetry may alter the dielectric response along the three crystallographic axes.

For 5,7-α_1_ and 5,7-α_2_ HBs, we found ε_*xx*_ > ε_*yy*_ > ε_*zz*_, indicating a clear anisotropy in the relative dielectric permittivity. This anisotropy suggests that charge carrier dynamics may differ along the principal crystallographic directions, which could influence the directional electrical response of these lattices. In a qualitative sense, larger dielectric screening along a given direction can be associated with enhanced carrier delocalization, and therefore, the carrier response may be more pronounced along the direction of a higher dielectric constant. A rigorous evaluation of carrier mobility would require explicit transport calculations and is beyond the scope of the present work. The observed dielectric anisotropy provides an indication of possible direction-dependent transport behavior.

Such directional trends may be consistent with previous reports on B_1_H_1_, where thermal conductivity was found to be larger along the zigzag direction than along the armchair direction (335 vs 293 W·m^–1^·K^–1^),^27^ suggesting anisotropic energy and charge transport in related borophane systems. These characteristics highlight the potential relevance of HB monolayers for anisotropic nanoelectronic or optoelectronic applications, where direction-dependent dielectric screening can influence device performance. Hong et al.^9^ previously reported that three-nanometer-thick amorphous boron nitride films exhibit ultralow dielectric constants of 1.78 and 1.16 at operating frequencies of 100 kHz and 1 MHz, respectively, in line with International Roadmap for Devices and Systems targets for high-performance electronics,^9,10,64^ underscoring the broader interest in low-κ anisotropic materials.

The anisotropic dielectric response can be traced to the bonding topology of the HB sheets. Hydrogen atoms form three-center, two-electron (3c, 2e) B–H–B bonds that are slightly displaced from the boron plane and distributed on both sides of the layer. The associated charge localization therefore extends differently along in-plane and out-of-plane directions, producing direction-dependent polarizability and distinct ε_*xx*_, ε_*yy*_, and ε_*zz*_ components. ELF analysis supports this interpretation by showing that the localization regions of the 3c–2e bonds extend anisotropically relative to the boron framework.

Figure 4 displays the PBEsol calculated total ε^*r*^ curves, which may represent the refraction of light,^64^ for each of the HBs, including that of the monolayer reported in ref 55. The zero-frequency point of each curve, ε(0), corresponds to the net static dielectric permittivity, which varies between 1.47 and 1.63 for the monolayers examined, displaying that ε(0) is smaller for 6,6 HB than for 5,7 HB (see individual and mean values of ε in Table 3). The dispersion of mean ε^*r*^ remains normal (nearly flat) up to 1.5 eV for all the 6,6 HBs, suggesting the semimetallic nature of the studied systems. The behavior is true regardless of the polarization type (shown only for 6,6 HB, Figure 4e), in which, ε^*r*^ gradually rises, peaking at approximately 2.4 eV before decaying, which is what is called abnormal dispersion (or abnormal scattering).^65^ The presence of prominent electronic screening effect is not noticeable in the infrared-to-visible region <2.5 eV since ε^*r*^ does not conceive any intense peak in this region, as observed for free-standing graphene.^1^

**Figure 4:**
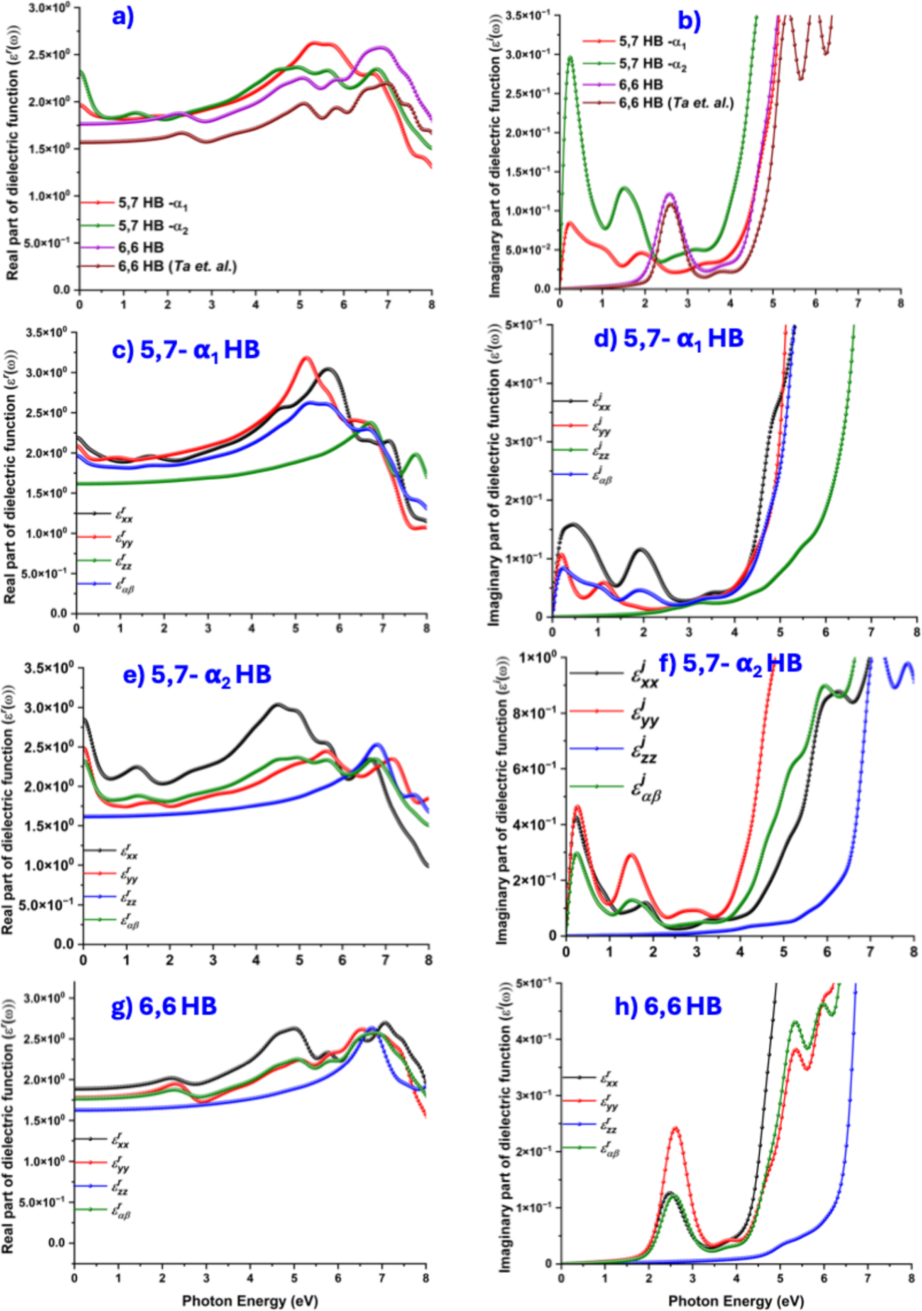
Illustration of the real and imaginary parts of the dielectric function (ε^*r*^ and ε^*i*^, respectively) as a function of photon energy. (a) Plot of the total ε^*r*^, calculated as ε^*r*^ = ε^*r*^_αβ_ = ^1^/_3_[ε^*r*^_*xx*_ + ε^*r*^_*yy*_ + ε^*r*^_*zz*_], for all three monolayers of 5–7 and 6–6 borophanes. (b) Plot of the total ε^*i*^, calculated as ε^*i*^ = ε^*i*^_αβ_ = ^1^/_3_[ε^*i*^_*xx*_ + ε^*i*^_*yy*_ + ε^*i*^_*zz*_], for all three monolayers of 5–7 and 6–6 borophanes; the *xx*, *yy*, *zz*, and total polarized spectra of real and imaginary parts of the dielectric function for (c,d) 5,7-α_1_, (e,f)­5,7-α_2_, and (g,h) 6,6-HB systems, respectively.

The ε^r^ curves for 6,6 HBs are not the same as those found for 5,7-borophanes. In the latter, a peak appears at zero energy, which is due to free electrons. This is followed by a sharp decline in the ε^*r*^ curve, clearly indicating relatively large metallicity^66^ of both 5,7-α_1_ and 5,7-α_2_ (5,7-α_2_ > 5,7-α_1_) This behavior is consistent across all three polarized curves along the three mutually perpendicular directions (see Figure 4e for 5,7-α_2_).

We did not observe any Drude-tail in the ε^r^ curve from the infrared (0 eV < photon energy <1.5 eV) though visible (1.5 eV < photon energy <3.5 eV) to the UV region (3.5 eV < photon energy <10 eV) of any of the studied lattices; this tail generally connects the positive (ε^*r*^ > 0) to negative (ε^r^ < 0) nature of the curve at the zero-crossing photon energy,^67^ unlike what is seen in other semimetallic 2D systems,^67^ e.g, BeP_2_,^46^ B_18_, and C_15_N_3_.^44^ However, our results may resemble those reported for N_18_, B_6_N_12_, and B_3_N_15_ ^44^ and are consistent with that reported for 6,6 HB.^27^ The aforementioned attribute is not unlikely due to the presence of both electron and hole conduction at the Fermi level (see Figure 2), which dampens the low-frequency response and diminishes the Drude-like behavior. The absence of Drude-tail in the energy region mentioned above does not completely rule out the existence of surface plasmons as such a feature was previously reported for semimetallic graphene^3^ and in low-dimensional transition metal dichalcogenides due to the excitonic effects. Borophene features plasmonic resonance to due to the large number of band crossings.^56^ The bulk and surface plasmonic phenomena generally emerge when ε^r^ = 0 and ε^r^ = −1, respectively.^67^ It has been suggested that 2D systems with high intrinsic carrier density may sustain plasmons in the visible range, which is particularly advantageous for optical devices,^59^ but this is not intense for the lattices examined.

We indeed detected a very weak Drude-like feature at the zero-crossing photon energies of 9.6 and 11.8 eV in *yy*- and *zz*-polarizations, respectively, for 6,6 HB (Figure S7). This was not found for the remaining monolayers investigated. This Drude feature is linked to modes responsible for plasmonic excitation, as the ε_*ii*_ curves exhibit poles (reflects resonant behavior) at the above energies,^64^ leading to a singularity in the dielectric response of the material, suggesting that conduction electrons oscillate with plasma frequencies could be in phase along the propagation length of the respective lattices. The detailed nature of the ε^*r*^ curves in the 0–45 eV energy range can be inferred from Figure S8.

The mean of the imaginary part of the complex dielectric function, ε^*i*^_αβ_, for each of the four lattices is plotted in Figure 2b, including the polarized contributions for all polytypes of 5,7 and 6,6 HBs in Figure 2d, f, and h, respectively. A notable characteristic of the ε^*i*^_αβ_ curve is the presence of critical points associated with light absorption. As can be seen, the ε^i^ curve shows two prominent peaks in the far- and near-infrared regions for both 5,7-α_1_ and 5,7-α_2_. This behavior is typical of semimetallic systems where low-energy intraband and interband transitions can produce finite optical response close to zero photon energy.^68^ The specific aspect was previously observed in different directions ([100], [010], and [001]) in the case of transition metal chalcogenides (e.g., Weyl semimetal WTe_2_ and MoTe_2_)^69^ as well as ZrTe_2_ and NiTe_2_ within the 0–2.5 eV energy range,^70^ yet this region was accompanied by an appreciable Drude tail in ε^*r*^.

The onset of optical absorption begins above 1.5 eV for 6,6 HB lattice, which qualitatively resembles the behavior observed in Al_2_O_3_; however, in Al_2_O_3_, this onset occurs around 6.0 eV.^71^ The anisotropy noted above for ε^*r*^_αβ_ (Figure 2c,e,g) is also observed in the ε^*i*^_αβ_ curves (Figure 2d,f,h). The intensity of the *zz*-polarized optical response of ε^*i*^_αβ_ is much weaker compared with that of the *xx*- and *yy*-polarizations. For 5,7-α_1_, the first three peaks in the ε^*i*^_αβ_ curve show up at photon energies of 0.2, 1.0, and 1.9 eV (Figure 2b). For 5,7-α_2_, the first three *xx*-polarized optical peaks are positioned at photon energies of 0.3 (0.3), 1.5 (1.8), and 2.9 (3.4) eV (Figure 2f), where the parentheses values represent the peak positions of the *yy*-polarized curve. The *zz*-polarized curve is nearly constant and rises above 4.3 eV, meaning that the in-plane polarization plays a key role in the absorption process. Such features are absent in the ε^*i*^_αβ_ curves in the same energy range.

For 6,6 HB, the optical absorptions are direct (vertical) interband transitions in which the momenta of initial and final states are equal, and no additional scattering is required,^66^ peaking in the 2.5–3.0 eV region. This behavior was previously noted for 6,6 HB (see Figure 4B of ref 20). The study demonstrated that the PBE computed optical absorption begins at 2.3 eV, while the zz-polarization occurs above 4.5 eV. This is indeed in agreement with the *xx*- and *yy*-polarization features that show absorption around 2.8 and 2.5 eV, respectively, whereas the *zz*-polarization shows a very weak shoulder-like attribute that peaks around 5.1 eV. These results do not contradict the fundamental definition of ε^*i*^_αβ_, in which the response curve should start from the photon energy of 0.0 eV, with subsequent absorptions in the infrared, visible, and UV–visible regions. Our results agree reasonably with the PBE ^27^and many-body G_0_W_0_-level^55^ study reported for 6,6 HB. There are very marginal differences in the precise positions of the optical absorption due to the varying level of exchange and correlation of each computational method chosen; none of these approaches has failed to predict that the ε^*i*^_αβ_ curve should accompany the onset of optical absorption above 2.0 eV. The study of Ta et al.^55^ suggests that the PBE (and G_0_W_0_@PBE)-level spectrum has primary (excitonic) absorption peaks within the violet region at 2.4 (3.0) and 2.2 (2.8) eV for the *xx*- and *yy-*polarization directions, respectively, contributing to the material’s yellow color.^20^ Moreover, ε^*i*^_αβ_ approaches near to zero at photon energies greater than 30 eV (Figure S9), suggesting that both 5,7 and 6,6 HB monolayers are likely to behave as transparent materials at these energies.^67^

The present work focuses on hydrogenated borophene phases. While pristine borophene polymorphs exhibit stronger metallic response, hydrogenation redistributes the electronic density through B–H–B multicenter bonding, leading to reduced low-energy absorption and enhanced dielectric anisotropy compared with fully metallic borophene sheets.

The absorption coefficient, α­(ω), is related to the dielectric function through the relationship given by $\alpha \left(\omega \right)=\frac{\surd 2\omega }{c}{\left[\sqrt{\left(\varepsilon_{1}^{2}+\varepsilon_{2}^{2}\right)}-\varepsilon_{1}^{2}\right]}^{1/2}$ . Using α­(ω), we have obtained the so-called Tauc plot shown in Figure 5. The nature of absorption of the two 5,7 HB polytypes is drastically different from that of 6,6 HB monolayers, especially in the energy-range between 0 and 1.8 eV. This region is not very flat for 6,6 HB but associated with the systematic rise of absorption. The subsequent onset of absorption is located between the energy range 1.8 and 2.1 eV for the three monolayers. We found that the first absorption peak is located at photon energies of 2.85 eV for the 6,6 HB. For the monolayer of the same system reported elsewhere,^55^ the corresponding absorption peak is calculated to be located at the photon energy of 2.65 eV. Our calculated result for 6,6 HB polymorphs may still be considered reasonable when compared with that of the experimentally observed onset of optical absorption (excitation peak) around 2.90 eV, which was inferred from the UV–vis spectra of 6,6 HB nanosheets (see Figure 3F in ref 20), and is somehow higher than the value of 2.25 eV reported as the optical bandgap of borophene.^5,72^ These results are also consistent with the molar absorption coefficient measurements reported by Hikichi et al.^73^ to further support the reliability of our calculated optical spectra. The calculated absorption maximum for 6,6 HB appears at ∼2.85 eV, which corresponds to ∼350 nm and agrees well with the reported experimental molar absorption peak near 350 nm for HB nanosheets. The optical onset in the calculations occurs at ∼2.1 eV, while experimental spectra show a slightly higher onset energy. This difference is expected because semilocal PBE functionals typically underestimate excitation energies. Nevertheless, the agreement in peak position and spectral shape supports the validity of the present electronic structure description.

**Figure 5:**
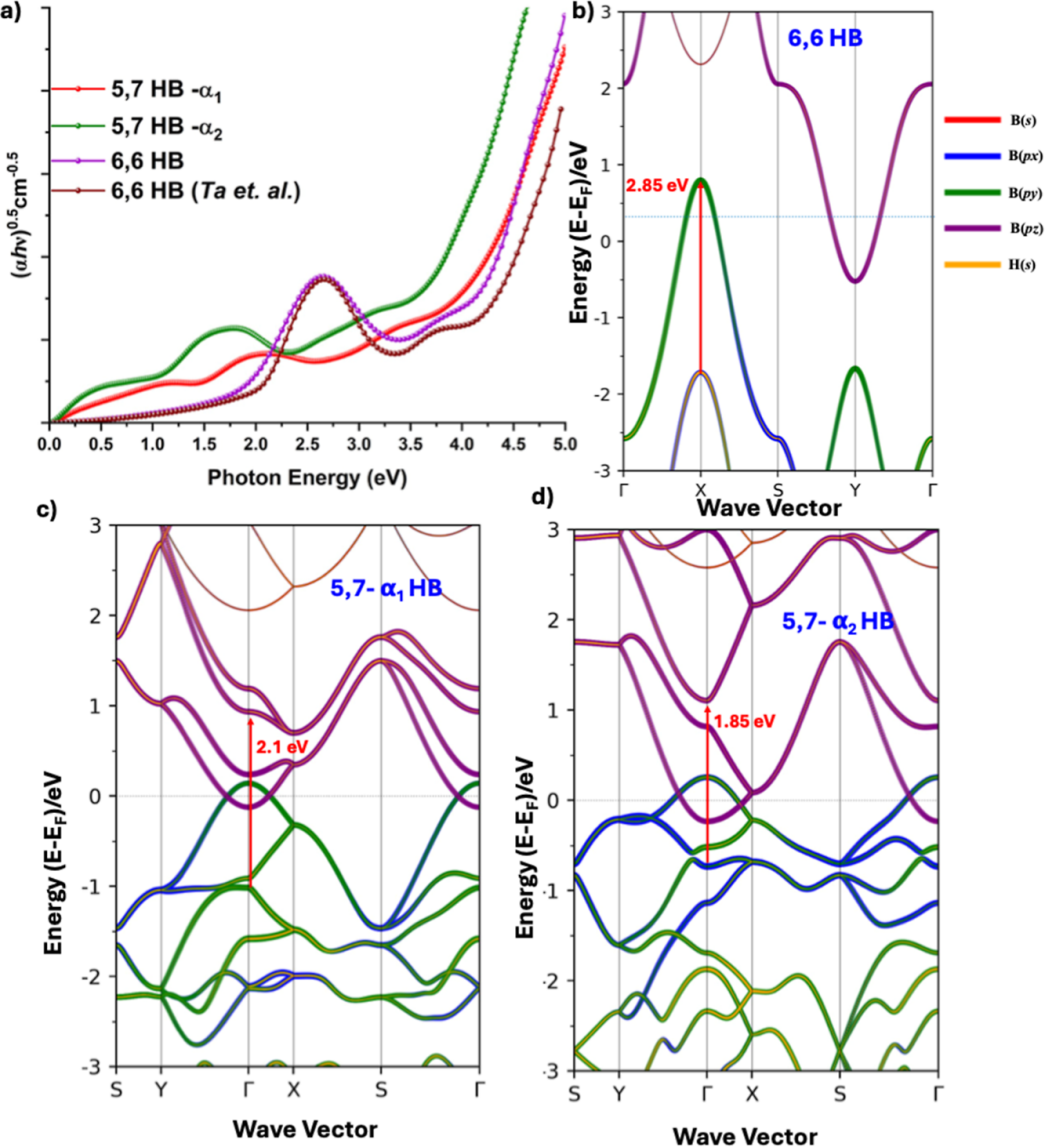
(a) Comparison of PBEsol-level Tauc plots for all the three HB systems in the photon energy range 0.0–4.6 eV. (b–d) Optical transitions are shown in PBEsol band structures of 6,6, 5,7-α_1_ and 5,7-α_2_ HBs respectively, as illustrated in (a).

In the 2.6–3.4 eV range, where the first absorption peak for 6,6 HBs occurs, there is no significant absorption observed for the two 5,7 monolayers; however, a gradual increase of the Tauc curve is evident, which extends dramatically into the UV region of the electromagnetic spectrum. For the 5,7 monolayers, two weak absorption features appear in the 0–2.4 eV range, specifically at photon energies of 1.1 eV (0.5) and 2.1 eV (1.85) for 5,7-α_1_ (5,7-α_2_), respectively, indicating that the light absorption capacity of 5,7-α_2_ should exceed that of 5,7-α_1_, which are also evidence of the dielectric function ε^*i*^_αβ_. Also, we can observe the rise of high absorption peaks in the 3.5–4 eV range for the 5,7 HB monolayers, indicating the presence of strong interband transitions that dominate the optical response at higher photon energies. The pronounced absorption in the 3.5–4 eV range suggests that 5,7 HB monolayers could serve as efficient materials for ultraviolet (UV) light absorption, with potential applications in UV photodetectors, UV-blocking coatings, or photocatalytic devices.

The dominant spectral features originate primarily from transitions between B-p-derived states. In the low-energy region, the absorption involves mainly in-plane *p*_*x*_/*p*_*y*_ states near the Fermi level, while higher-energy features arise from transitions to antibonding states with mixed p character. Although a full transition density matrix (TDM) or excitonic analysis is beyond the scope of the present work, the orbital-resolved density of states and band structures provides a consistent interpretation of the main spectral features. Excitonic effects and local-field corrections are not included within the present independent-particle treatment and may introduce small shifts in peak positions but are not expected to alter the qualitative anisotropic trends discussed here.

## 4 Conclusions

In this study, we have systematically investigated the lattice, electronic, and optical properties of hydrogenated borophene, focusing on three polymorphs of the 5,7, and 6,6 HB monolayers. The calculated real and imaginary parts of dielectric functions reveal that the 5,7 HB structures exhibit a stronger low-energy optical response, consistent with their semimetallic electronic features and the presence of low-energy electronic excitations in the far-infrared region. The optical absorption spectra derived from the complex dielectric function further highlight how symmetry and hydrogenation patterns modify the electronic transitions across these polymorphs. Overall, our results clarify the relationship between structure, bonding, and optical behavior in hydrogenated borophene and provide useful design guidelines for engineering two-dimensional boron-based materials for applications in infrared photonics, tunable optoelectronics, and energy-related devices. The observed polarization-dependent dielectric response and tunable absorption across the infrared–visible range suggest that hydrogenated borophene could serve as a model platform for exploring anisotropic two-dimensional optoelectronic materials, although detailed transport and device-level studies will be required to assess practical implementation.

## Supplementary Material



## Notes

Phonon dispersion information obtained from DFPT calculations for the 5,7-α1, 5,7-α2, and 6,6 HB monolayers; ab initio molecular dynamics (AIMD) simulations at 300, 500, and 700 K demonstrating thermal stability; projected density of states and electronic band structure comparisons at the PBEsol level; a representative comparison of PBEsol and SCAN *meta*-GGA band structures; additional real and imaginary parts of the dielectric function over extended photon energy ranges; and tables summarizing the optimized lattice parameters and bonding characteristics of the PBEsol structures (PDF)
