# Supplementary material for: Dielectric Response of Hydrogenated Borophene Monolayers from First-Principles Density Functional Theory Calculations
Source: ACS Omega. 2026 Apr 6;11(15):23007–19. doi: 10.1021/acsomega.5c12691 (PMC13103752; doi:10.1021/acsomega.5c12691)
Supplement: Supplementary file 1 [file ao5c12691_si_001.pdf]

Dielectric Response of Hydrogenated Borophene Monolayers from First-Principles Density Functional Theory Calculations

Arpita Varadwaj[1*], Yasunobu Ando[2], Masahito Niibe[3], Takahiro Kondo[4], Iwao Matsuda[3] and Masato Kotsugi[1]

Faculty of Advanced Engineering, Tokyo University of Science, Tokyo 125-8585, Japan,

[2] Institute of Integrated Research, Institute of Science Tokyo, Yokohama, Kanagawa, 226-8501, Japan

[3] The Institute for Solid State Physics, The University of Tokyo, Kashiwa, Chiba 277-8581, Japan,

[4]Institute of Pure and Applied Sciences, University of Tsukuba, Tsukuba, Ibaraki, 305-8573, Japan

*Corresponding author's e-mail: varadwaj.arpita@gmail.com

Electronic Supplementary Information (ESI)

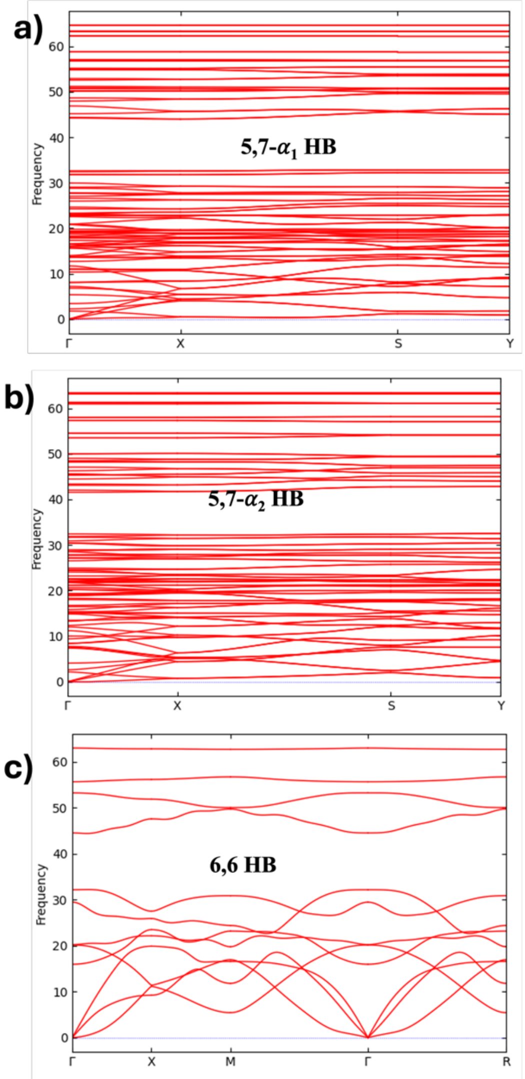

Fig. S1: Phonon band structures of the hydrogenated borophene (HB) monolayers obtained from DFPT calculations: (a) 5,7-α1 HB, (b) 5,7-α2 HB and (c) 6,6 HB

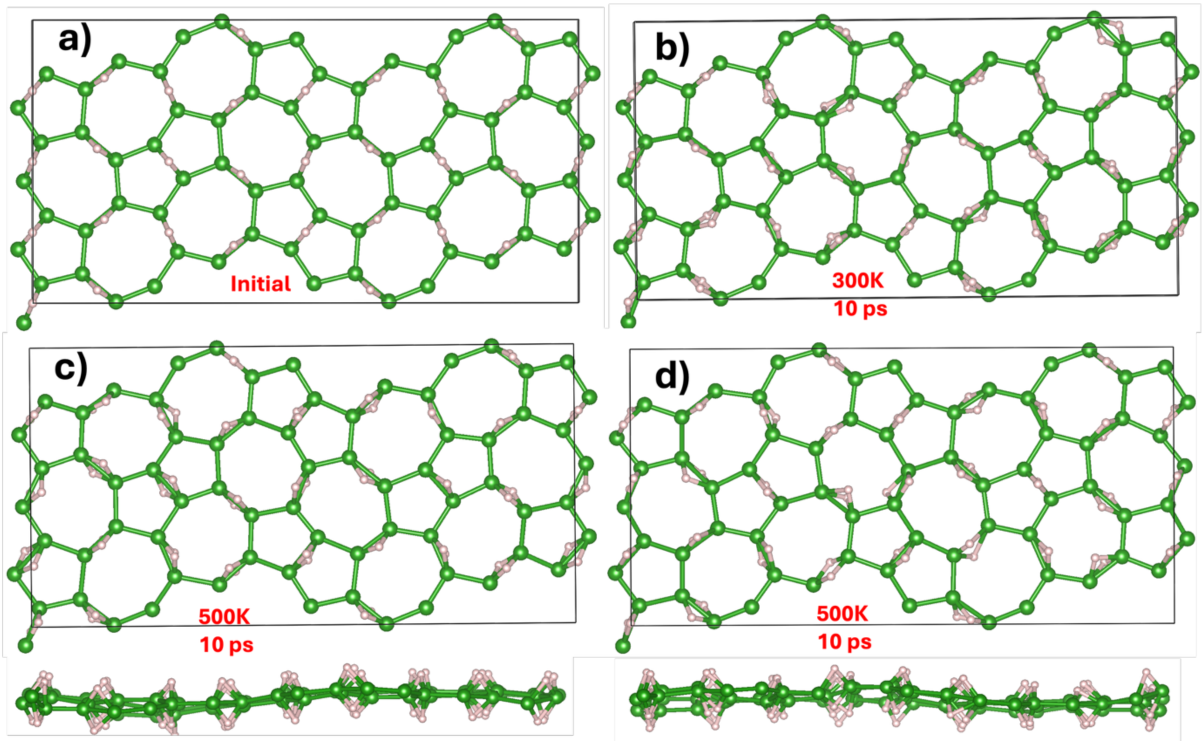

Fig. S2. Ab initio molecular dynamics (AIMD) simulations of the 5,7-α_1 HB monolayer. (a) Initial configuration. Final structures after 10 ps NVT simulations at (b) 300 K, (c) 500 K, and (d) 700 K are shown. Top views are provided for all temperatures, while both top and side views are shown for 500 K and 700 K to illustrate the stability of the B–H–B framework at elevated temperatures.

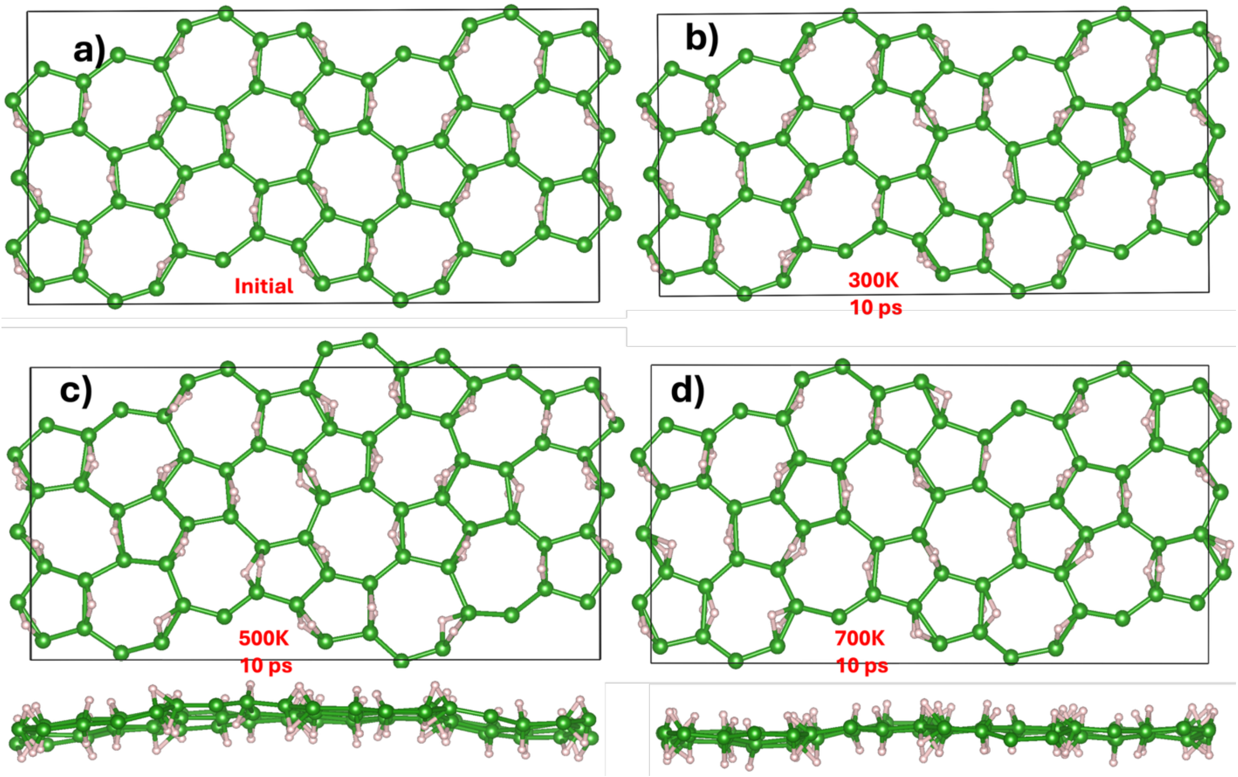

Fig. S3. Ab initio molecular dynamics (AIMD) simulations of the 5,7-α_1 HB monolayer. (a) Initial configuration. Final structures after 10 ps NVT simulations at (b) 300 K, (c) 500 K, and (d) 700 K are shown. Top views are provided for all temperatures, while both top and side views are shown for 500 K and 700 K to illustrate the stability of the B–H–B framework at elevated temperatures.

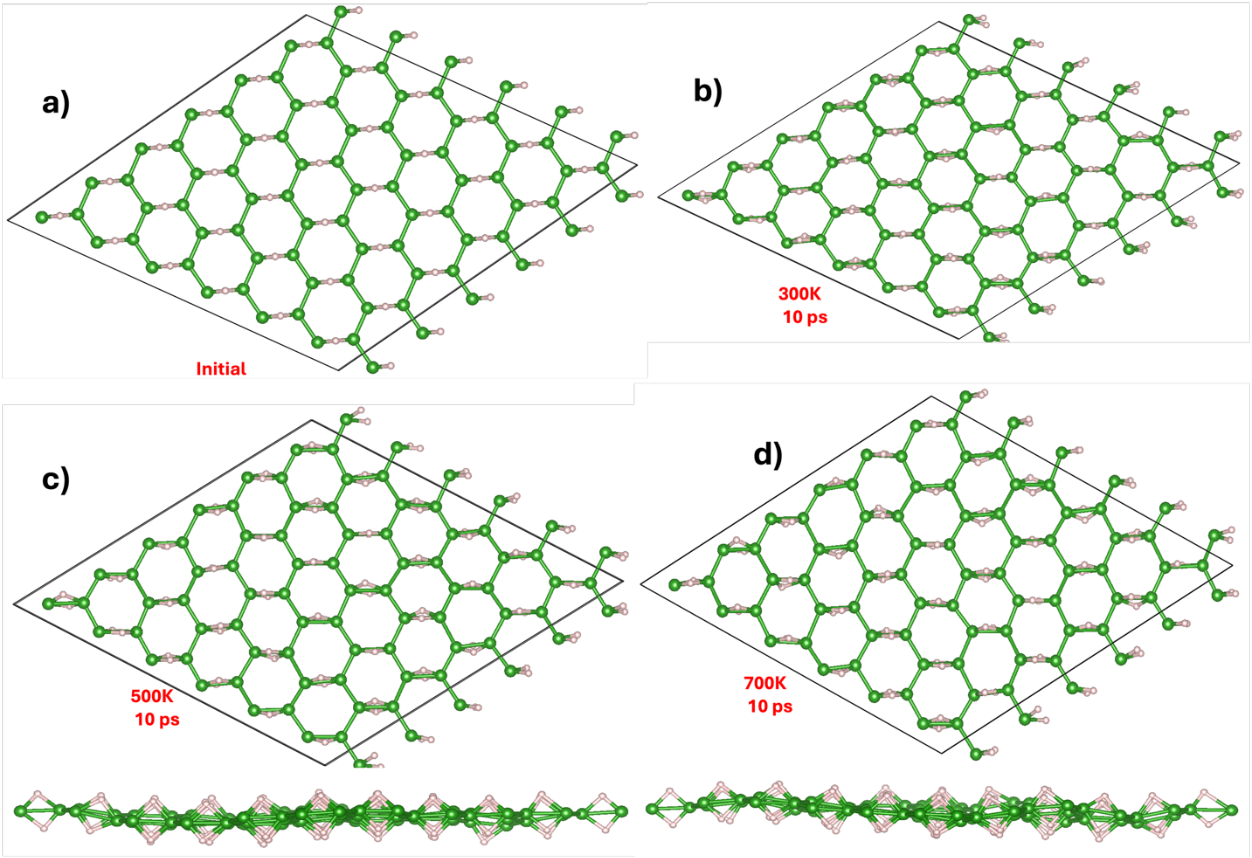

Fig. S4. Ab initio molecular dynamics (AIMD) simulations of the 6,6 HB monolayer. (a) Initial configuration. Final structures after 10 ps NVT simulations at (b) 300 K, (c) 500 K, and (d) 700 K are shown. Top views are provided for all temperatures, while both top and side views are shown for 500 K and 700 K to illustrate the stability of the B–H–B framework at elevated temperatures.

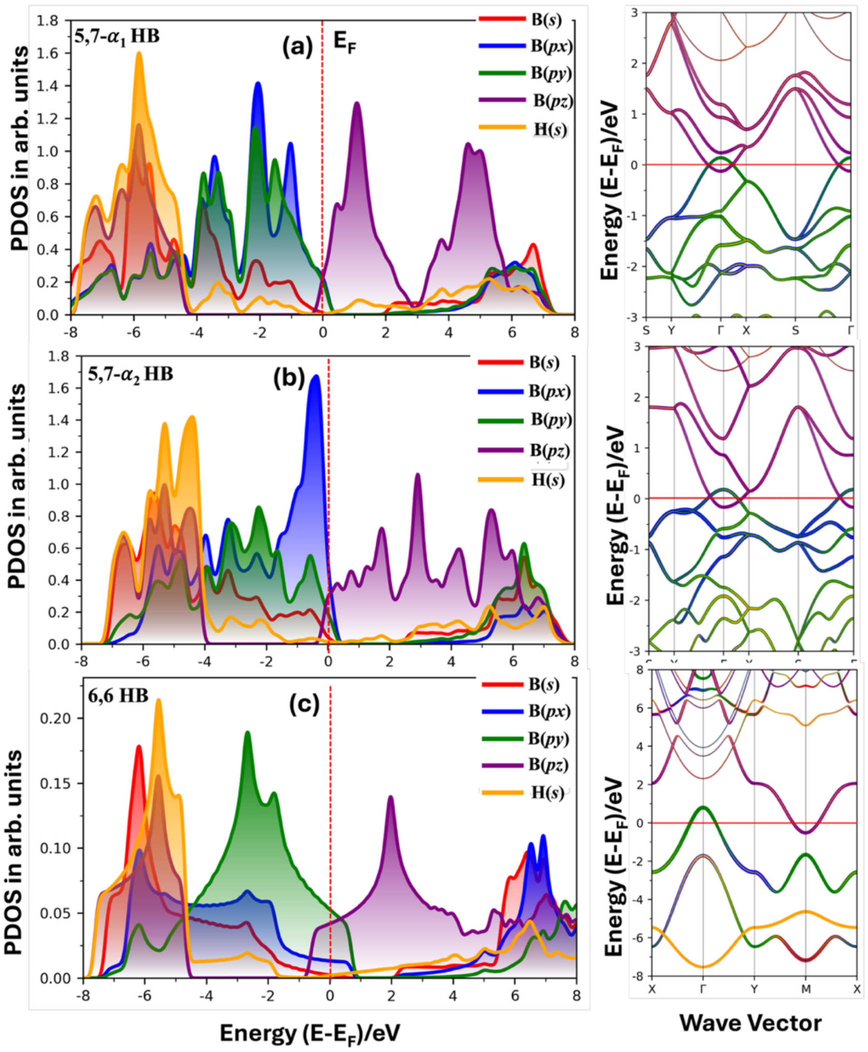

Fig. S5: Comparison of PBEsol-level (left) projected density of states (PDOS) and (right) electronic band structure of (a) 5,7-α_1, (b) 5,7-α_2, (c) 6,6-HB (Cmmm), Fermi level, E_F, marked at 0.0 eV in (a) is also applicable to (b)-(c). The color code used for PDOS for each atom (and orbital) type is the same as that of the band structure.

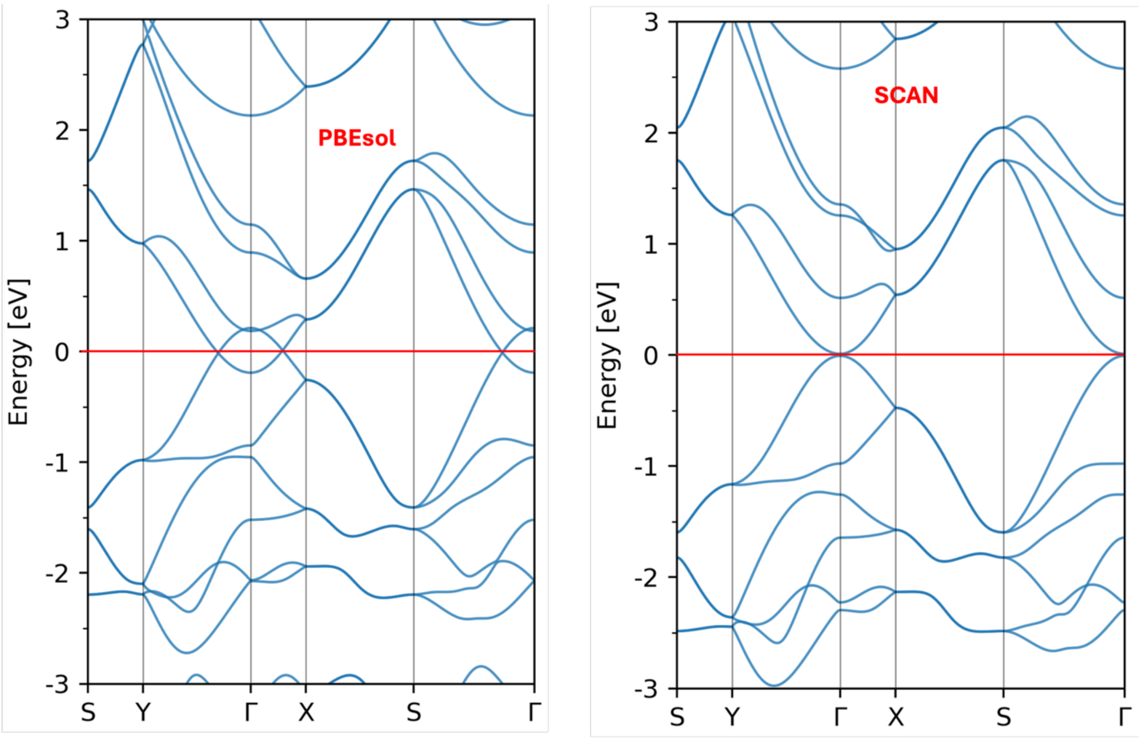

Fig. S6: A representative comparison of the PBEsol and SCAN band structures is provided in the Supporting Information

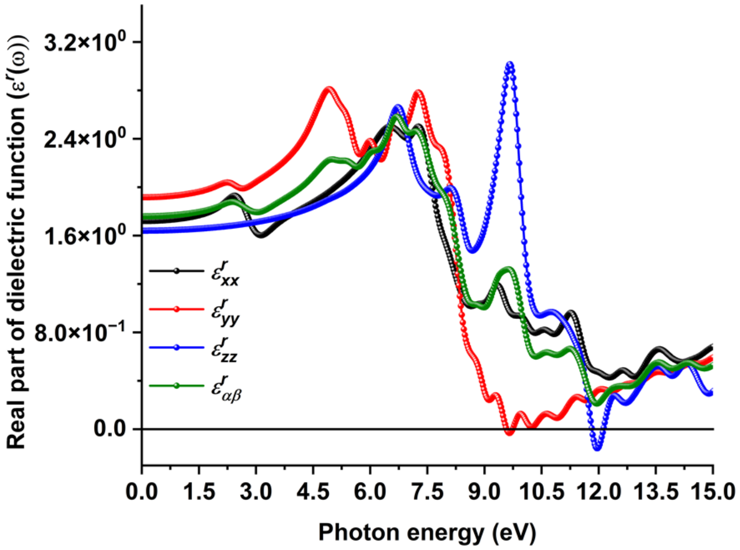

Fig. S7: PBEsol-level illustration of the real part of the dielectric function as a function of photon energy for 6,6 HB in the range of 0 to 15 eV, highlighting the presence of a Drude feature.

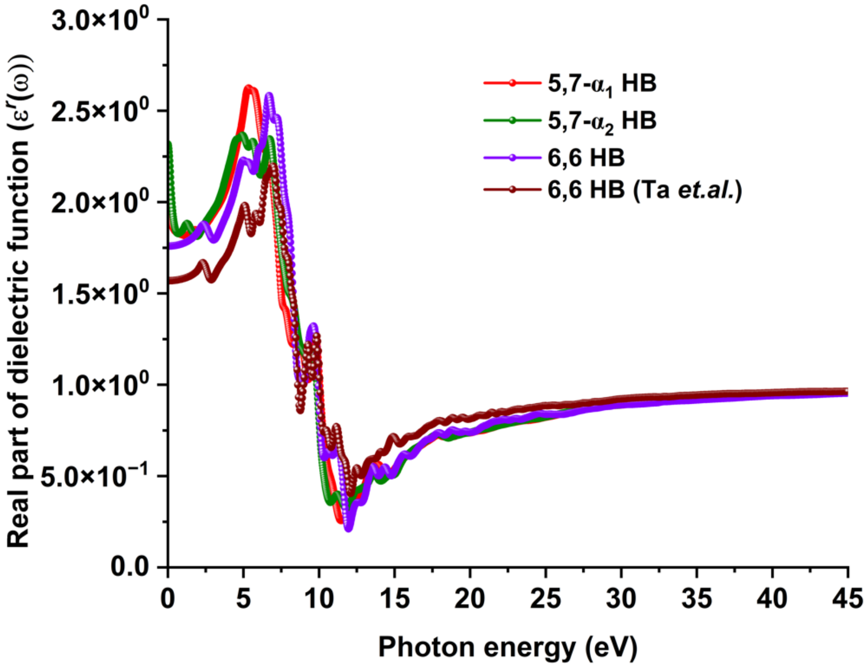

Fig. S8: PBEsol-level illustration of the real part of the dielectric function as a function of photon energy for all three monolayers of HB, covering the range from 0 to 45 eV.

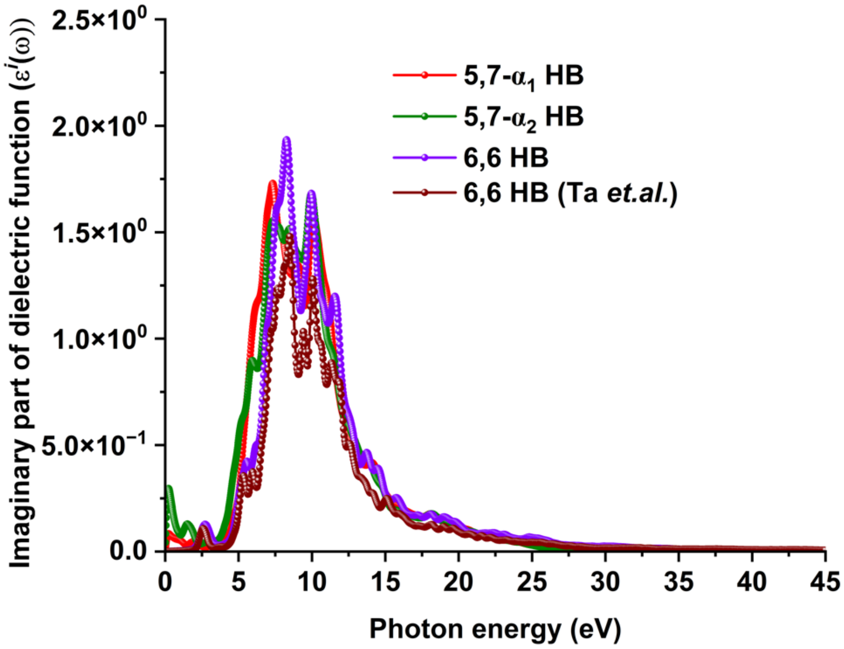

Fig. S9: PBEsol-level illustration of the imaginary part of the dielectric function as a function of photon energy for all three monolayers of HB, covering the range from 0 to 45 eV.

Table S1: PBEsol optimized lattice constants (a, b, α, β, and γ), and energy/atom for different polymorphs of 5,7- and 6,6 HB monolayers.

Crystal system (space group)	Formula unit	*a* (Å)	*b* (Å)	*α* (°)	*β* (°)	*γ* (°)	Energy/atom (eV)
5,7-α_1 HB	$B_{16}H_{16}$	11.295	5.820	90	90	90.0	-4.90133
5,7-α_2 HB	$B_{16}H_{16}$	11.300	5.810	90	90	90.0	-4.92313
6,6 HB	B_2H_2	3.046	3.046	90	90	120.6	-4.96280

Table S2: Selected PBEsol level average intranuclear atom-atom bond distances (Å) and bond angles (degrees) of the polymorphs of 5-7 and 6-6 HB monolayers.

System	Boron ring type	*r*(B-B)	*r*(B-B)$_b$	*r*(B-H)	∠B-B-B	∠B-H-B
5-7 HB-α_1	B_5-ring	1.745±0.018	1.827±0.0	1.322±0.0	108.0±2.1	87.4±0.0
	B_7-ring	1.732±0.029	1.788±0.029	1.325 ± 0.009	128.6 ± 8.8	84.8 ± 0.9
5-7 HB-α_2	B_5-ring	1.718±0.025	1.794±0.059	1.339±0.011	108.0±5.1	85.1±3.2
	B_7-ring	1.727±0.014	1.794±0.059	1.339±0.011	128.6 ± 5.7	84.3 ± 2.6
6,6 HB	B_6-ring	1.714±0.0	1.809±0.0	1.328±0.0	120.3 ±1.3	85.8±0.0
